# Supplementary material for: Monitoring the Microevolution of Salmonella enterica in Healthy Dairy Cattle Populations at the Individual Farm Level Using Whole-Genome Sequencing
Source: Front Microbiol. 2021 Oct 18;12:763669. doi: 10.3389/fmicb.2021.763669 (PMC8558520; doi:10.3389/fmicb.2021.763669)
Supplement: Supplementary file 6 [file Data_Sheet_1.PDF]

Supplementary Text. Extended version of the Materials and Methods presented in the main manuscript.

1 Supplementary Materials and Methods

1.1 Isolate Selection

Salmonella enterica isolates (*n* = 128) obtained from one of 13 dairy farms in New York State were selected to undergo WGS for this study (Supplementary Table S1). All strains were isolated from farms that had undergone surveillance for *Salmonella* for a period of at least 12 months as described previously (Cummings et al., 2010; Rodriguez-Rivera et al., 2014). Strains were isolated from repeated sampling on each farm between October 2007 and August 2009, from either (i) fecal samples from healthy, subclinically infected dairy cows (referred to hereafter as "bovine" isolates), or (ii) farm environmental swabs (referred to hereafter as "farm environmental" isolates) (Cummings et al., 2010). All isolates underwent serotyping, phenotypic antimicrobial susceptibility testing, and pulsed-field gel electrophoresis (PFGE) as described previously (Rodriguez-Rivera et al., 2014).

1.2 Whole-Genome Sequencing and Data Pre-Processing

Genomic DNA extraction and sequencing library preparation were performed as described previously (Carroll et al., 2017b), and the genomes of all 128 *Salmonella* isolates were sequenced using an Illumina HiSeq platform and 2 x 250 bp paired-end reads. Illumina sequencing adapters and low-quality bases were trimmed using Trimmomatic version 0.33 (using default parameters for Nextera paired-end reads) (Bolger et al., 2014), and FastQC version 0.11.9 (Andrews, 2019) was used to confirm adapter removal and assess read quality. SPAdes version 3.8.0 (Bankevich et al., 2012) was used to assemble genomes *de novo* (using the "careful" option and *k*-mer sizes of 21, 33, 55, 77, 99, and 127), and QUAST version 4.5 (Gurevich et al., 2013) and the "lineage_wf" workflow implemented in CheckM version 1.1.3 (Parks et al., 2015) were used to assess the quality of the resulting assemblies. MultiQC version 1.8 (Ewels et al., 2016) was used to aggregate genome quality metrics. Genome quality statistics are available for all isolates (Supplementary Table S1).

1.3 *In Silico* Serotyping

In addition to undergoing traditional serotyping in a laboratory setting (i.e., serological detection of expressed O and H antigens using the White-Kauffmann-Le Minor scheme) as described previously (Rodriguez-Rivera et al., 2014), all 128 assembled *Salmonella* genomes (see section "Whole-Genome Sequencing and Data Pre-Processing" above) underwent *in silico* serotyping using the command line implementations of (i) the *Salmonella In Silico* Typing Resource (SISTR) version 1.0.2 (Yoshida et al., 2016) and (ii) SeqSero2 version 1.1.1 (Zhang et al., 2019) (using SeqSero2's *k*-mer based workflow). Serotypes assigned using all three methods are available for all 128 isolates (Supplementary Table S1). In cases where a discrepancy existed among the traditional serotype designation and one or more of the *in silico* methods, the serotype assigned using two out of the three methods was selected as the final serotype to be reported (e.g., when assigning strain names to isolates in the manuscript, for phylogeny annotation). To confirm that all serotype assignments were reasonable, a phylogeny was constructed using core

single nucleotide polymorphisms (SNPs) detected in all *Salmonella* genomes in this study (see section "Reference-Free SNP Identification and Phylogeny Construction" below).

1.4 *In Silico* Antimicrobial Resistance Determinant Detection

Antimicrobial resistance (AMR) determinants were detected in each of the 128 *Salmonella* genomes using five separate approaches: (i) ABRicate (https://github.com/tseemann/abricate) version 0.8 (Seemann, 2018), (ii) AMRFinderPlus version 3.2.3 (Feldgarden et al., 2019), (iii) ARIBA version 2.14.1 (Hunt et al., 2017), (iv) BTyper version 2.3.3 (Carroll et al., 2017a), and (v) SRST2 version 0.2.0 (Inouye et al., 2014). Assembled genomes were used as input for the ABRicate and BTyper approaches, while trimmed Illumina reads were used as input for the SRST2 and ARIBA approaches. Prokka version 1.12 (Seemann, 2014) was used to annotate each assembled genome, and the resulting GFF (.gff) and FASTA (.faa and .ffn) files were used as input for the AMRFinderPlus approach. For the ABRicate approach, the following AMR gene databases were tested (each accessed June 11, 2018 via ABRicate's abricate-get_db command): (i) the Antibiotic Resistance Gene-ANNOTation database (ARG-ANNOT) (Gupta et al., 2014), (ii) the Comprehensive Antibiotic Resistance Database (CARD) (Jia et al., 2017), (iii) the National Center for Biotechnology Information's (NCBI's) Bacterial Antimicrobial Resistance Reference Gene Database (NCBI) (Feldgarden et al., 2019), and (iv) the ResFinder database (ResFinder) (Zankari et al., 2012). For each genome and database combination, minimum AMR gene identity and coverage thresholds ranging from 50-100% (5% increments) and 0-100% (10% increments) were tested, respectively. For the BTyper approach, the (i) ARG-ANNOT v3 and (ii) MEGARes version 1.0.1 (Lakin et al., 2017) databases available with BTyper version 2.3.3 were used, with the minimum AMR gene identity and coverage thresholds varied in a manner identical to the ABRicate approach. For the SRST2 approach, the (i) ARG-ANNOT and (ii) ResFinder databases available with SRST2 version 0.2.0 were tested, using default thresholds. For the ARIBA approach, the following databases were tested (each accessed June 13, 2019 using ARIBA's getref command): (i) the version of ARG-ANNOT available with SRST2, (ii) CARD, (iii) MEGARes, (iv) NCBI, and (v) ResFinder, with all default thresholds used. For the AMRFinder approach, the latest version of the AMRFinder database was used (accessed December 6, 2019), along with the organism-specific database for *Salmonella*.

1.5 *In Silico* Prediction of Antimicrobial Minimum Inhibitory Concentration Values

The PATRIC3 antimicrobial minimum inhibitory concentration (MIC) prediction model for *Salmonella* (Nguyen et al., 2019) (accessed June 13, 2019) was used to predict MIC values for each of the 128 *Salmonella* isolates in this study, using the assembled genome of each as input. The following were used as dependencies: kmc version 3.0 (Kokot et al., 2017) and XGBoost version 0.82 (Chen and Guestrin, 2016).

1.6 Prediction of Phenotypic Susceptible-Intermediate-Resistant Classifications Using *In Silico* Methods

All 128 *Salmonella* isolates underwent phenotypic antimicrobial susceptibility testing with a panel of 15 antimicrobials (i.e., amikacin, amoxicillin-clavulanic acid, ampicillin, cefoxitin, ceftiofur, ceftriaxone, chloramphenicol, ciprofloxacin, gentamicin, kanamycin, nalidixic acid, streptomycin, sulfamethoxazole-trimethoprim, sulfisoxazole, and tetracycline) using the

Sensititre® system (Trek Diagnostic Systems Ltd., Cleveland, OH) available at Cornell University's Animal Health Diagnostic Center as described previously (Rodriguez-Rivera et al., 2014). A "true" (i.e., phenotypic) susceptible-intermediate-resistant (SIR) classification for each of the 15 antimicrobials was obtained for 126 *Salmonella* isolates by comparing raw MIC values to NARMS breakpoints for *Salmonella* (accessed March 23, 2020; Supplementary Table S1). For streptomycin, the 1996-2013 NARMS breakpoints were used, as this was compatible with the concentrations used at the time of phenotypic testing (Rodriguez-Rivera et al., 2014). For sulfisoxazole, isolates with MIC > 256 were classified as resistant, as a concentration of 512 μg/mL was not tested. While raw MIC values were unavailable for two isolates (BOV_KENT_16_04-03-08_R8-0967 and ENV_MELA_01_01-10-08_R8-0165; Supplementary Table S1), both isolates had previously been categorized as pan-susceptible to all 15 antimicrobials (a classification that was maintained here, as all *in silico* methods correctly classified these isolates as pan-susceptible).

Supplemental Table S4 of the AMRFinder validation paper (Feldgarden et al., 2019) was used to identify known AMR determinant/phenotype associations for AMR determinants detected by each of the AMR determinant detection pipeline/database combinations described above (see section "*In Silico* Antimicrobial Resistance Determinant Detection") and all of the 15 antimicrobials tested in this study (Supplementary Table S2). If a detected AMR gene was not identified in the AMRFinder literature search, the gene name was queried in CARD, and the resulting literature linked to the CARD entry was searched for known AMR genotype/phenotype associations (Supplementary Table S2). An isolate was predicted to be resistant to a particular antimicrobial if it possessed one or more AMR determinants known to confer resistance to that antimicrobial; if it did not possess any AMR determinants known to confer resistance to that antimicrobial, the isolate was predicted to be susceptible to that antimicrobial (Supplementary Table S2). For each AMR determinant detection pipeline/database combination, the caret package (Kuhn, 2008) in R version 3.6.1 (R Core Team, 2019) was used to construct a confusion matrix and calculate accuracy scores, Cohen's kappa coefficients, and other statistics (Supplementary Table S3) by treating "true" susceptible/resistant classifications obtained using phenotypic susceptibility testing as a reference. Cases of intermediate phenotypic resistance were treated as susceptible, as it resulted in slightly higher accuracy scores for all pipeline/database combinations for this particular data set. Because *in silico* prediction of susceptibility/resistance was highly dependent on prior knowledge of AMR determinants and the antimicrobials to which they conferred resistance, the concordance of all pipeline/database combinations was assessed by comparing each pipeline/database combination to results obtained using the SRST2 pipeline/ARG-ANNOT database combination.

To assess the ability of the MIC prediction method implemented in PATRIC3 to predict *Salmonella* SIR classification (see section "*In Silico* Prediction of Antimicrobial Minimum Inhibitory Concentration Values" above), predicted MIC values for 14 antimicrobials produced using PATRIC3 were used to predict the SIR status of each of the 128 *Salmonella* isolates using the same NARMS breakpoints used for phenotypic testing. Azithromycin MICs produced by PATRIC3 were excluded, as azithromycin was not among the 15 antimicrobials used here for phenotypic testing. The ability of PATRIC3 to predict amikacin resistance was also not evaluated, as amikacin is not among the antimicrobials queried by PATRIC3. A confusion matrix was constructed as described above, using predicted SIR classifications derived from predicted MIC values produced by PATRIC3 and NARMS breakpoints. Additionally, the deviation of raw MIC predictions produced by PATRIC3 ($MIC_{PATRIC3}$) from "true" raw MIC

predictions produced using phenotypic testing ($MIC_{Phenotypic}$) in number of dilution factors ($N_{dilution\ factors}$) was assessed using the following equation:

$$N_{dilution\ factors} = \frac{\ln\left(\frac{MIC_{PATRIC3}}{MIC_{Phenotypic}}\right)}{\ln(2)}$$

where ln corresponds to the natural logarithm. For example: if PATRIC3 predicted an MIC value of 8 and the "true" MIC value obtained with phenotypic testing was 2, then ln(8/2)/ln(2) = 2; this means that the PATRIC3 prediction of 8 is +2 dilution factors away from the "true" MIC of 2 (as dilution used for MIC are 2 fold serial dilutions, e.g., 2 µg/mL, 4 µg/mL, 8 µg/mL).

1.7 Re-testing of Isolates with Highly Incongruent AMR Phenotypes

Several (n = 21) isolates possessed a phenotypic AMR SIR profile which was deemed to be highly incongruent with its predicted *in silico* AMR profile, regardless of the *in silico* pipeline/database used (Supplementary Table S4). For example, *S.* Cerro isolate BOV_CERO_35_10−02−08_R8−2685 was resistant to nine antimicrobials but did not harbor any known acquired AMR genes (Supplementary Table S4). Similarly, *S.* Newport isolate ENV_NEWP_62_03−05−09_R8−3442 itself was pan-susceptible, but harbored multiple acquired AMR genes (e.g., bla_{CMY-2}, *floR*, *sul2*, *tetA*), which conferred multidrug resistance in closely related *S.* Newport isolates (Supplementary Table S4). To address these incongruencies, 21 selected *Salmonella* isolates underwent phenotypic antimicrobial susceptibility re-testing (conducted September 16, 2020) as described above (see section "Prediction of Phenotypic Susceptible-Intermediate-Resistant Classifications Using *In Silico* Methods"), with the exception of amikacin and kanamycin, as the contemporary panel did not include these antimicrobials (Supplementary Table S4).

Kanamycin testing was conducted separately using a gradient diffusion assay (Jorgensen and Ferraro, 2009) according to the manufacturer's instructions (BioMérieux Kanamycin Strip KM 256, product number 412381). Briefly, bacterial isolates were streaked for single colonies onto Brain Heart Infusion (BHI, Becton Dickinson [BD], Franklin Lakes, NJ, USA) agar plates from frozen glycerol stocks. Precultures were prepared by inoculating a single colony in 3 mL Mueller-Hinton (MH) broth (BD Difco), followed by incubating at 37°C with shaking at 200 rpm for 12-14h. The precultures were used to inoculate tubes with 5 mL MH broth at 1:200 dilution, and the tubes were incubated at 37°C with shaking at 200 rpm for 5 hours. Four mL of melted MH soft agar medium (0.7% agar) were mixed with 100 µL of culture and poured onto Petri plates containing 15 mL of MH agar medium (0.7% agar), and the plates were dried for 5 min. Kanamycin gradient strips were laid on top of the soft agar, and the plates were incubated at 35°C for 18 hours. MIC values were determined by evaluating the inhibition zone using a magnifying lens according to the manufacturer's instructions.

MIC values obtained from re-testing these isolates were interpreted within NARMS breakpoints as described above (see section "Prediction of Phenotypic Susceptible-Intermediate-Resistant Classifications Using *In Silico* Methods") and are reported in the main manuscript (with the exception of amikacin; due to its exclusion from the contemporary panel, original MIC values are reported). Original and updated MIC and SIR values for all 21 isolates are available in Supplementary Table S4.

1.8 *In Silico* Plasmid Replicon Detection

Plasmid replicons were detected in all *Salmonella* genome assemblies using ABRicate and the PlasmidFinder database (accessed June 11, 2018 via ABRicate's abricate-get_db command). For a plasmid replicon to be considered present in a genome, minimum nucleotide BLAST (blastn) (Camacho et al., 2009) identity and coverage values of 80 and 60%, respectively, were used (Carattoli et al., 2014).

1.9 Reference-Free SNP Identification and Phylogeny Construction

A reference-free approach was used to compare the 128 *Salmonella* genomes sequenced in this study to 442 of the 445 *Salmonella* genomes described by Worley et al. (Worley et al., 2018); three genomes were omitted because their Sequence Read Archive (SRA) data was not publicly available at the time of access (February 20, 2019). Raw reads for each of the 442 publicly available genomes were downloaded from SRA (Leinonen et al., 2011; Kodama et al., 2012) and processed and assembled as described above (see section "Whole-Genome Sequencing and Data Pre-Processing" described above). kSNP3 version 3.1 (Gardner and Hall, 2013; Gardner et al., 2015) was used to identify core SNPs among all 570 assembled *Salmonella* genomes, using the optimal *k*-mer size determined by Kchooser ($k = 19$). IQ-TREE version 1.6.10 (Nguyen et al., 2015) was used to construct a maximum likelihood (ML) phylogeny using the resulting core SNPs and the optimal nucleotide substitution model identified using ModelFinder (determined using model Bayesian Information Criteria [BIC] values); the optimal model was the transversion model with unequal, empirical base frequencies, an ascertainment bias correction, and the FreeRate model with six categories, i.e., the TVM +F+ASC+R6 model (Yang, 1995; Lewis, 2001; Soubrier et al., 2012; Kalyaanamoorthy et al., 2017). Bootstrapping was performed using 1,000 replicates of the Ultrafast Bootstrap method (Minh et al., 2013; Hoang et al., 2018). The resulting ML phylogeny was annotated in R using the bactaxR package (Carroll et al., 2020b) and the following dependencies: ape (Paradis et al., 2004; Paradis and Schliep, 2019), dplyr (Wickham et al., 2020), ggtree (Yu et al., 2017; Yu et al., 2018), phylobase (R Hackathon, 2019), phytools (Revell, 2012), and reshape2 (Wickham, 2007).

1.10 Pan-Genome Characterization

GFF files produced by Prokka (see section "*In Silico* Antimicrobial Resistance Determinant Detection" above) were used as input for Roary version 3.12.0 (Page et al., 2015), which was used to identify orthologous gene clusters at a 70% protein BLAST (blastp) identity threshold. The resulting gene presence/absence matrix produced by Roary was used as input for besPLOT (https://github.com/lmc297/besPLOT) (Carroll et al., 2020a), which was used to perform non-metric multidimensional scaling (NMDS) (Kruskal, 1964) and construct plots in two dimensions using a Jaccard distance metric and the following dependencies in R: vegan version 2.5-6 (Oksanen et al., 2019), shiny version 1.4.0.2 (Chang et al., 2020), ggplot2 version 3.3.0 (Wickham, 2016), plyr version 1.8.6 (Wickham, 2011), dplyr version 0.8.5 (Wickham et al., 2020), cluster version 2.1.0 (Maechler et al., 2019), and ggrepel version 0.8.2 (Slowikowski, 2020).

Clustering based on gene presence/absence was assessed for each of the following grouping factors: (i) serotype, (ii) farm, and (iii) isolation source (i.e., bovine or farm environmental). For each of the three grouping factors, the following three statistical tests were performed, using the gene presence/absence matrix produced by Roary, a Jaccard distance

metric, and 10,000 permutations: (i) the permutest and betadisper functions in R's vegan package (Oksanen et al., 2019) were used to conduct an ANOVA-like permutation test (Anderson, 2006) to test if group dispersions were homogenous (referred to hereafter as the PERMDISP2 test); (ii) analysis of similarity (ANOSIM) (Clarke, 1993) using the anosim function in the vegan package in R was used to determine if the average of the ranks of within-group distances was greater than or equal to the average of the ranks of between-group distances (Anderson and Walsh, 2013); (iii) permutational analysis of variance (PERMANOVA) (Anderson, 2001) using the adonis2 function in the vegan package in R was used to determine if group centroids were equivalent. For all tests, a Bonferroni correction was applied to correct for multiple comparisons.

Potential clustering based on AMR gene presence/absence was additionally assessed for the same three grouping factors (serotype, farm, and isolation source), using the presence and absence of AMR determinants detected by AMRFinderPlus as input (i.e., AMR and stress response determinants identified using the "plus" option in AMRFinderPlus). All steps were performed as described above, and a Bonferroni correction was used to correct for multiple comparisons.

1.11 Reference-Based Core SNP Identification Within Serotypes

For each individual serotype, core SNPs were identified among genomes assigned to that serotype using a reference-based approach. For each serotype, Snippy version 4.3.6 (https://github.com/tseemann/snippy) (Seemann, 2019b) was used to identify core SNPs among all representatives assigned to the serotype, using the trimmed Illumina paired-end reads of each genome as input (see section "Whole-Genome Sequencing and Data Pre-Processing" above), one of six high-quality assembled genomes from isolates in this study as a reference genome (Supplementary Table S1), and the following dependencies: BWA MEM version 0.7.13-r1126 (Li and Durbin, 2009; Li, 2013), Minimap2 version 2.15 (Li, 2018), SAMtools version 1.8 (Li et al., 2009), BEDtools version 2.27.1 (Quinlan and Hall, 2010; Quinlan, 2014), BCFtools version 1.8 (Li, 2011), FreeBayes version v1.1.0-60-gc15b070 (Garrison and Marth, 2012), vcflib version v1.0.0-rc2 (Cleary et al., 2015), vt version 0.57721 (Tan et al., 2015), SnpEff version 4.3T (Cingolani et al., 2012), samclip version 0.2 (Seemann, 2019a), seqtk version 1.2-r102-dirty (Li, 2019), and snp-sites version 2.4.0 (Page et al., 2016). Gubbins version 2.3.4 (Croucher et al., 2015) was used to identify and remove recombination within the full alignment that resulted, and the filtered alignment produced by Gubbins was queried using snp-sites to produce an alignment of core SNPs for each serotype.

1.12 Construction of Within-Serotype Phylogenies

For each serotype, IQ-TREE version 1.6.10 was used to construct a ML phylogeny, using core SNPs detected among all isolates assigned to the serotype as input (see "Reference-Based Core SNP Identification Within Serotypes" section above), the optimal ascertainment bias-aware nucleotide substitution model selected using ModelFinder, and 1,000 replicates of the UltraFast bootstrap approximation. The temporal structure of each resulting ML phylogeny was assessed using the R^2 value produced by the best-fitting root in TempEst version 1.5.1 (Supplementary Table S5) (Rambaut et al., 2016).

A tip-dated phylogeny was then constructed for each serotype using BEAST version 2.5.0 (Bouckaert et al., 2014; Bouckaert et al., 2019), the appropriate core SNP alignment, an initial clock rate of 2.79×10^{-7} substitutions/site/year (Leekitcharoenphon et al., 2016), and an

ascertainment bias correction to account for the use of solely variant sites (Bouckaert, 2014). The modelTest function in R's phangorn package (Schliep, 2011) was used to select an appropriate nucleotide substitution model for each serotype, and the implementation of the model with the lowest BIC value in BEAST 2's SSN package (Bouckaert and Xie, 2017) was selected. For each serotype, combinations of either a strict or lognormal relaxed molecular clock (Drummond et al., 2006) and either a coalescent constant or coalescent Bayesian Skyline (Drummond et al., 2005) population model were tested. For all models, a lognormal prior was used for the clockRate/ucldMean parameters (in real space, M = 2.058×10^{-6} and S = 2.0, yielding a median value of 2.79×10^{-7} substitutions/site/year, and 2.5% and 97.5% quantiles of 5.53×10^{-9} and 1.40×10^{-5} substitutions/site/year, respectively). For each molecular clock and population model combination for each serotype, three independent stepping stone sampling analyses (Xie et al., 2011) were performed with BEAST 2, using 10 steps of at least 100 million generations. Bayes factors (Kass and Raftery, 1995) were calculated to determine the optimal combination of molecular clock and population model for each serotype (Supplementary Table S5). Ten independent BEAST 2 runs were then performed using the optimal model for each serotype, using chain lengths of at least 100 million generations, sampling every 10,000 generations. Tracer version 1.7.1 (Rambaut et al., 2018) was used to ensure adequate mixing of each independent run. The resulting log and tree files were aggregated using LogCombiner-2, and TreeAnnotator-2 (Heled and Bouckaert, 2013) was used to produce a maximum clade credibility tree using 10% burn-in. The phylogenies were annotated using R and the following packages: ggplot2, ggtree, and phylobase. Bayesian skyline plots for all serotype groups analyzed using BEAST2 are also available (Supplementary Figure S1); however, due to the limited number of available isolates surveyed among each serotype and the short temporal range queried here, potential changes in effective population sizes may not be robust and are thus not discussed in this study.

1.13 Data Availability

Illumina reads are available for all isolates sequenced in this study under NCBI Bioproject Accession PRJNA756552. NCBI BioSample accession numbers for each individual isolate, as well as all associated metadata and genome quality statistics, are available in Supplementary Table S1. All BEAST 2 XML files used for temporal phylogeny construction are available at https://github.com/lmc297/zru_farms.

2 Supplementary References

Anderson, M.J. (2001). A new method for non-parametric multivariate analysis of variance. *Austral Ecology* 26**,** 32-46.

Anderson, M.J. (2006). Distance-Based Tests for Homogeneity of Multivariate Dispersions. *Biometrics* 62**,** 245-253.

Anderson, M.J., and Walsh, D.C.I. (2013). PERMANOVA, ANOSIM, and the Mantel test in the face of heterogeneous dispersions: What null hypothesis are you testing? *Ecological Monographs* 83**,** 557-574.

Andrews, S. (2019). "FastQC: a quality control tool for high throughput sequence data". 0.11.8 ed.

Bankevich, A., Nurk, S., Antipov, D., Gurevich, A.A., Dvorkin, M., Kulikov, A.S., Lesin, V.M., Nikolenko, S.I., Pham, S., Prjibelski, A.D., Pyshkin, A.V., Sirotkin, A.V., Vyahhi, N., Tesler, G., Alekseyev, M.A., and Pevzner, P.A. (2012). SPAdes: a new genome assembly algorithm and its applications to single-cell sequencing. *J Comput Biol* 19**,** 455-477.

Bolger, A.M., Lohse, M., and Usadel, B. (2014). Trimmomatic: a flexible trimmer for Illumina sequence data. *Bioinformatics* 30**,** 2114-2120.

Bouckaert, R. (2014). *Correcting for constant sites in BEAST2* [Online]. Available: https://groups.google.com/forum/#!topic/beast-users/QfBHMOqImFE [Accessed 25 September 2021].

Bouckaert, R., Heled, J., Kuhnert, D., Vaughan, T., Wu, C.H., Xie, D., Suchard, M.A., Rambaut, A., and Drummond, A.J. (2014). BEAST 2: a software platform for Bayesian evolutionary analysis. *PLoS Comput Biol* 10**,** e1003537.

Bouckaert, R., Vaughan, T.G., Barido-Sottani, J., Duchene, S., Fourment, M., Gavryushkina, A., Heled, J., Jones, G., Kuhnert, D., De Maio, N., Matschiner, M., Mendes, F.K., Muller, N.F., Ogilvie, H.A., Du Plessis, L., Popinga, A., Rambaut, A., Rasmussen, D., Siveroni, I., Suchard, M.A., Wu, C.H., Xie, D., Zhang, C., Stadler, T., and Drummond, A.J. (2019). BEAST 2.5: An advanced software platform for Bayesian evolutionary analysis. *PLoS Comput Biol* 15**,** e1006650.

Bouckaert, R., and Xie, D. (2017). "SSN: Standard Nucleotide Substitution Models".

Camacho, C., Coulouris, G., Avagyan, V., Ma, N., Papadopoulos, J., Bealer, K., and Madden, T.L. (2009). BLAST+: architecture and applications. *BMC Bioinformatics* 10**,** 421.

Carattoli, A., Zankari, E., Garcia-Fernandez, A., Voldby Larsen, M., Lund, O., Villa, L., Moller Aarestrup, F., and Hasman, H. (2014). *In silico* detection and typing of plasmids using PlasmidFinder and plasmid multilocus sequence typing. *Antimicrob Agents Chemother* 58**,** 3895-3903.

Carroll, L.M., Huisman, J.S., and Wiedmann, M. (2020a). Twentieth-century emergence of antimicrobial resistant human- and bovine-associated *Salmonella enterica* serotype Typhimurium lineages in New York State. *Sci Rep* 10**,** 14428.

Carroll, L.M., Kovac, J., Miller, R.A., and Wiedmann, M. (2017a). Rapid, High-Throughput Identification of Anthrax-Causing and Emetic *Bacillus cereus* Group Genome Assemblies via BTyper, a Computational Tool for Virulence-Based Classification of *Bacillus cereus* Group Isolates by Using Nucleotide Sequencing Data. *Appl Environ Microbiol* 83.

Carroll, L.M., Wiedmann, M., Den Bakker, H., Siler, J., Warchocki, S., Kent, D., Lyalina, S., Davis, M., Sischo, W., Besser, T., Warnick, L.D., and Pereira, R.V. (2017b). Whole-Genome Sequencing of Drug-Resistant *Salmonella enterica* Isolates from Dairy Cattle

and Humans in New York and Washington States Reveals Source and Geographic Associations. *Appl Environ Microbiol* 83.
Carroll, L.M., Wiedmann, M., and Kovac, J. (2020b). Proposal of a Taxonomic Nomenclature for the *Bacillus cereus* Group Which Reconciles Genomic Definitions of Bacterial Species with Clinical and Industrial Phenotypes. *mBio* 11**,** e00034-00020.
Chang, W., Cheng, J., Allaire, J., Xie, Y., and Mcpherson, J. (2020). "shiny: Web Application Framework for R. R package version 1.4.0.2. https://CRAN.R-project.org/package=shiny".
Chen, T., and Guestrin, C. (2016). "XGBoost: A Scalable Tree Boosting System", in: *Proceedings of the 22nd ACM SIGKDD International Conference on Knowledge Discovery and Data Mining.* (San Francisco, California, USA: Association for Computing Machinery).
Cingolani, P., Platts, A., Wang Le, L., Coon, M., Nguyen, T., Wang, L., Land, S.J., Lu, X., and Ruden, D.M. (2012). A program for annotating and predicting the effects of single nucleotide polymorphisms, SnpEff: SNPs in the genome of *Drosophila melanogaster* strain w1118; iso-2; iso-3. *Fly (Austin)* 6**,** 80-92.
Clarke, K.R. (1993). Non-parametric multivariate analyses of changes in community structure. *Australian Journal of Ecology* 18**,** 117-143.
Cleary, J.G., Braithwaite, R., Gaastra, K., Hilbush, B.S., Inglis, S., Irvine, S.A., Jackson, A., Littin, R., Rathod, M., Ware, D., Zook, J.M., Trigg, L., and De La Vega, F.M. (2015). Comparing Variant Call Files for Performance Benchmarking of Next-Generation Sequencing Variant Calling Pipelines. *bioRxiv***,** 023754.
Croucher, N.J., Page, A.J., Connor, T.R., Delaney, A.J., Keane, J.A., Bentley, S.D., Parkhill, J., and Harris, S.R. (2015). Rapid phylogenetic analysis of large samples of recombinant bacterial whole genome sequences using Gubbins. *Nucleic Acids Res* 43**,** e15.
Cummings, K.J., Warnick, L.D., Elton, M., Grohn, Y.T., Mcdonough, P.L., and Siler, J.D. (2010). The effect of clinical outbreaks of salmonellosis on the prevalence of fecal *Salmonella* shedding among dairy cattle in New York. *Foodborne Pathog Dis* 7**,** 815-823.
Drummond, A.J., Ho, S.Y., Phillips, M.J., and Rambaut, A. (2006). Relaxed phylogenetics and dating with confidence. *PLoS Biol* 4**,** e88.
Drummond, A.J., Rambaut, A., Shapiro, B., and Pybus, O.G. (2005). Bayesian coalescent inference of past population dynamics from molecular sequences. *Mol Biol Evol* 22**,** 1185-1192.
Ewels, P., Magnusson, M., Lundin, S., and Kaller, M. (2016). MultiQC: summarize analysis results for multiple tools and samples in a single report. *Bioinformatics* 32**,** 3047-3048.
Feldgarden, M., Brover, V., Haft, D.H., Prasad, A.B., Slotta, D.J., Tolstoy, I., Tyson, G.H., Zhao, S., Hsu, C.H., Mcdermott, P.F., Tadesse, D.A., Morales, C., Simmons, M., Tillman, G., Wasilenko, J., Folster, J.P., and Klimke, W. (2019). Validating the AMRFinder Tool and Resistance Gene Database by Using Antimicrobial Resistance Genotype-Phenotype Correlations in a Collection of Isolates. *Antimicrob Agents Chemother* 63.
Gardner, S.N., and Hall, B.G. (2013). When whole-genome alignments just won't work: kSNP v2 software for alignment-free SNP discovery and phylogenetics of hundreds of microbial genomes. *PLoS One* 8**,** e81760.

Gardner, S.N., Slezak, T., and Hall, B.G. (2015). kSNP3.0: SNP detection and phylogenetic analysis of genomes without genome alignment or reference genome. *Bioinformatics* 31**,** 2877-2878.

Garrison, E., and Marth, G. (2012). Haplotype-based variant detection from short-read sequencing. *arXiv***,** 1207.3907.

Gupta, S.K., Padmanabhan, B.R., Diene, S.M., Lopez-Rojas, R., Kempf, M., Landraud, L., and Rolain, J.M. (2014). ARG-ANNOT, a new bioinformatic tool to discover antibiotic resistance genes in bacterial genomes. *Antimicrob Agents Chemother* 58**,** 212-220.

Gurevich, A., Saveliev, V., Vyahhi, N., and Tesler, G. (2013). QUAST: quality assessment tool for genome assemblies. *Bioinformatics* 29**,** 1072-1075.

Heled, J., and Bouckaert, R.R. (2013). Looking for trees in the forest: summary tree from posterior samples. *BMC Evol Biol* 13**,** 221.

Hoang, D.T., Chernomor, O., Von Haeseler, A., Minh, B.Q., and Vinh, L.S. (2018). UFBoot2: Improving the Ultrafast Bootstrap Approximation. *Mol Biol Evol* 35**,** 518-522.

Hunt, M., Mather, A.E., Sanchez-Buso, L., Page, A.J., Parkhill, J., Keane, J.A., and Harris, S.R. (2017). ARIBA: rapid antimicrobial resistance genotyping directly from sequencing reads. *Microb Genom* 3**,** e000131.

Inouye, M., Dashnow, H., Raven, L.A., Schultz, M.B., Pope, B.J., Tomita, T., Zobel, J., and Holt, K.E. (2014). SRST2: Rapid genomic surveillance for public health and hospital microbiology labs. *Genome Med* 6**,** 90.

Jia, B., Raphenya, A.R., Alcock, B., Waglechner, N., Guo, P., Tsang, K.K., Lago, B.A., Dave, B.M., Pereira, S., Sharma, A.N., Doshi, S., Courtot, M., Lo, R., Williams, L.E., Frye, J.G., Elsayegh, T., Sardar, D., Westman, E.L., Pawlowski, A.C., Johnson, T.A., Brinkman, F.S., Wright, G.D., and Mcarthur, A.G. (2017). CARD 2017: expansion and model-centric curation of the comprehensive antibiotic resistance database. *Nucleic Acids Res* 45**,** D566-D573.

Jorgensen, J.H., and Ferraro, M.J. (2009). Antimicrobial susceptibility testing: a review of general principles and contemporary practices. *Clin Infect Dis* 49**,** 1749-1755.

Kalyaanamoorthy, S., Minh, B.Q., Wong, T.K.F., Von Haeseler, A., and Jermiin, L.S. (2017). ModelFinder: fast model selection for accurate phylogenetic estimates. *Nat Methods* 14**,** 587-589.

Kass, R.E., and Raftery, A.E. (1995). Bayes Factors. *Journal of the American Statistical Association* 90**,** 773-795.

Kodama, Y., Shumway, M., Leinonen, R., and International Nucleotide Sequence Database, C. (2012). The Sequence Read Archive: explosive growth of sequencing data. *Nucleic Acids Res* 40**,** D54-56.

Kokot, M., Dlugosz, M., and Deorowicz, S. (2017). KMC 3: counting and manipulating k-mer statistics. *Bioinformatics* 33**,** 2759-2761.

Kruskal, J.B. (1964). Nonmetric multidimensional scaling: A numerical method. *Psychometrika* 29**,** 115-129.

Kuhn, M. (2008). Building Predictive Models in R Using the caret Package. *2008* 28**,** 26.

Lakin, S.M., Dean, C., Noyes, N.R., Dettenwanger, A., Ross, A.S., Doster, E., Rovira, P., Abdo, Z., Jones, K.L., Ruiz, J., Belk, K.E., Morley, P.S., and Boucher, C. (2017). MEGARes: an antimicrobial resistance database for high throughput sequencing. *Nucleic Acids Res* 45**,** D574-D580.

Leekitcharoenphon, P., Hendriksen, R.S., Le Hello, S., Weill, F.X., Baggesen, D.L., Jun, S.R., Ussery, D.W., Lund, O., Crook, D.W., Wilson, D.J., and Aarestrup, F.M. (2016). Global Genomic Epidemiology of *Salmonella enterica* Serovar Typhimurium DT104. *Appl Environ Microbiol* 82**,** 2516-2526.

Leinonen, R., Sugawara, H., Shumway, M., and International Nucleotide Sequence Database, C. (2011). The sequence read archive. *Nucleic Acids Res* 39**,** D19-21.

Lewis, P.O. (2001). A likelihood approach to estimating phylogeny from discrete morphological character data. *Syst Biol* 50**,** 913-925.

Li, H. (2011). A statistical framework for SNP calling, mutation discovery, association mapping and population genetical parameter estimation from sequencing data. *Bioinformatics* 27**,** 2987-2993.

Li, H. (2013). Aligning sequence reads, clone sequences and assembly contigs with BWA-MEM. *arXiv***,** 1303.3997.

Li, H. (2018). Minimap2: pairwise alignment for nucleotide sequences. *Bioinformatics* 34**,** 3094-3100.

Li, H. (2019). "Seqtk: a fast and lightweight tool for processing sequences in the FASTA or FASTQ format". 1.2-r102-dirty ed.

Li, H., and Durbin, R. (2009). Fast and accurate short read alignment with Burrows-Wheeler transform. *Bioinformatics* 25**,** 1754-1760.

Li, H., Handsaker, B., Wysoker, A., Fennell, T., Ruan, J., Homer, N., Marth, G., Abecasis, G., Durbin, R., and Genome Project Data Processing, S. (2009). The Sequence Alignment/Map format and SAMtools. *Bioinformatics* 25**,** 2078-2079.

Maechler, M., Rousseeuw, P., Struyf, A., Hubert , M., and Hornik, K. (2019). "cluster: Cluster Analysis Basics and Extensions". 2.1.0 ed.

Minh, B.Q., Nguyen, M.A., and Von Haeseler, A. (2013). Ultrafast approximation for phylogenetic bootstrap. *Mol Biol Evol* 30**,** 1188-1195.

Nguyen, L.T., Schmidt, H.A., Von Haeseler, A., and Minh, B.Q. (2015). IQ-TREE: a fast and effective stochastic algorithm for estimating maximum-likelihood phylogenies. *Mol Biol Evol* 32**,** 268-274.

Nguyen, M., Long, S.W., Mcdermott, P.F., Olsen, R.J., Olson, R., Stevens, R.L., Tyson, G.H., Zhao, S., and Davis, J.J. (2019). Using Machine Learning To Predict Antimicrobial MICs and Associated Genomic Features for Nontyphoidal *Salmonella*. *Journal of Clinical Microbiology* 57**,** e01260-01218.

Oksanen, J., Blanchet, F.G., Friendly, M., Kindt, R., Legendre, P., Mcglinn, D., Minchin, P.R., O'hara, R.B., Simpson, G.L., Solymos, P., Stevens, M.H.H., Szoecs, E., and Wagner, H. (2019). "vegan: Community Ecology Package. R package version 2.5-6. https://CRAN.R-project.org/package=vegan".

Page, A.J., Cummins, C.A., Hunt, M., Wong, V.K., Reuter, S., Holden, M.T., Fookes, M., Falush, D., Keane, J.A., and Parkhill, J. (2015). Roary: rapid large-scale prokaryote pan genome analysis. *Bioinformatics* 31**,** 3691-3693.

Page, A.J., Taylor, B., Delaney, A.J., Soares, J., Seemann, T., Keane, J.A., and Harris, S.R. (2016). SNP-sites: rapid efficient extraction of SNPs from multi-FASTA alignments. *Microb Genom* 2**,** e000056.

Paradis, E., Claude, J., and Strimmer, K. (2004). APE: Analyses of Phylogenetics and Evolution in R language. *Bioinformatics* 20**,** 289-290.

Paradis, E., and Schliep, K. (2019). ape 5.0: an environment for modern phylogenetics and evolutionary analyses in R. *Bioinformatics* 35**,** 526-528.
Parks, D.H., Imelfort, M., Skennerton, C.T., Hugenholtz, P., and Tyson, G.W. (2015). CheckM: assessing the quality of microbial genomes recovered from isolates, single cells, and metagenomes. *Genome Res* 25**,** 1043-1055.
Quinlan, A.R. (2014). BEDTools: The Swiss-Army Tool for Genome Feature Analysis. *Curr Protoc Bioinformatics* 47**,** 11 12 11-34.
Quinlan, A.R., and Hall, I.M. (2010). BEDTools: a flexible suite of utilities for comparing genomic features. *Bioinformatics* 26**,** 841-842.
R Core Team (2019). "R: A Language and Environment for Statistical Computing". 3.6.1 ed. (Vienna, Austria: R Foundation for Statistical Computing).
R Hackathon (2019). "phylobase: Base Package for Phylogenetic Structures and Comparative Data". 0.8.6 ed.
Rambaut, A., Drummond, A.J., Xie, D., Baele, G., and Suchard, M.A. (2018). Posterior Summarization in Bayesian Phylogenetics Using Tracer 1.7. *Syst Biol* 67**,** 901-904.
Rambaut, A., Lam, T.T., Max Carvalho, L., and Pybus, O.G. (2016). Exploring the temporal structure of heterochronous sequences using TempEst (formerly Path-O-Gen). *Virus Evol* 2**,** vew007.
Revell, L.J. (2012). phytools: an R package for phylogenetic comparative biology (and other things). *Methods in Ecology and Evolution* 3**,** 217-223.
Rodriguez-Rivera, L.D., Wright, E.M., Siler, J.D., Elton, M., Cummings, K.J., Warnick, L.D., and Wiedmann, M. (2014). Subtype analysis of *Salmonella* isolated from subclinically infected dairy cattle and dairy farm environments reveals the presence of both human- and bovine-associated subtypes. *Vet Microbiol* 170**,** 307-316.
Schliep, K.P. (2011). phangorn: phylogenetic analysis in R. *Bioinformatics* 27**,** 592-593.
Seemann, T. (2014). Prokka: rapid prokaryotic genome annotation. *Bioinformatics* 30**,** 2068-2069.
Seemann, T. (2018). "ABRicate: Mass screening of contigs for antimicrobial resistance or virulence genes".
Seemann, T. (2019a). "samclip: Filter SAM file for soft and hard clipped alignments". 0.2 ed.
Seemann, T. (2019b). "Snippy: Rapid haploid variant calling and core genome alignment". 4.3.6 ed.
Slowikowski, K. (2020). "ggrepel: Automatically Position Non-Overlapping Text Labels with 'ggplot2'. R package version 0.8.2. https://CRAN.R-project.org/package=ggrepel".
Soubrier, J., Steel, M., Lee, M.S.Y., Der Sarkissian, C., Guindon, S., Ho, S.Y.W., and Cooper, A. (2012). The Influence of Rate Heterogeneity among Sites on the Time Dependence of Molecular Rates. *Molecular Biology and Evolution* 29**,** 3345-3358.
Tan, A., Abecasis, G.R., and Kang, H.M. (2015). Unified representation of genetic variants. *Bioinformatics* 31**,** 2202-2204.
Wickham, H. (2007). Reshaping Data with the reshape Package. *2007* 21**,** 20.
Wickham, H. (2011). The Split-Apply-Combine Strategy for Data Analysis. *2011* 40**,** 29.
Wickham, H. (2016). *ggplot2: Elegant Graphics for Data Analysis.* Springer-Verlag New York.
Wickham, H., François, R., Henry, L., and Müller, K. (2020). "dplyr: A Grammar of Data Manipulation". 0.8.5 ed.

Worley, J., Meng, J., Allard, M.W., Brown, E.W., and Timme, R.E. (2018). Salmonella enterica Phylogeny Based on Whole-Genome Sequencing Reveals Two New Clades and Novel Patterns of Horizontally Acquired Genetic Elements. *MBio* 9.

Xie, W., Lewis, P.O., Fan, Y., Kuo, L., and Chen, M.H. (2011). Improving marginal likelihood estimation for Bayesian phylogenetic model selection. *Syst Biol* 60**,** 150-160.

Yang, Z. (1995). A space-time process model for the evolution of DNA sequences. *Genetics* 139**,** 993-1005.

Yoshida, C.E., Kruczkiewicz, P., Laing, C.R., Lingohr, E.J., Gannon, V.P., Nash, J.H., and Taboada, E.N. (2016). The Salmonella In Silico Typing Resource (SISTR): An Open Web-Accessible Tool for Rapidly Typing and Subtyping Draft Salmonella Genome Assemblies. *PLoS One* 11**,** e0147101.

Yu, G., Lam, T.T., Zhu, H., and Guan, Y. (2018). Two Methods for Mapping and Visualizing Associated Data on Phylogeny Using Ggtree. *Mol Biol Evol* 35**,** 3041-3043.

Yu, G., Smith, D.K., Zhu, H., Guan, Y., and Lam, T.T.-Y. (2017). ggtree: an r package for visualization and annotation of phylogenetic trees with their covariates and other associated data. *Methods in Ecology and Evolution* 8**,** 28-36.

Zankari, E., Hasman, H., Cosentino, S., Vestergaard, M., Rasmussen, S., Lund, O., Aarestrup, F.M., and Larsen, M.V. (2012). Identification of acquired antimicrobial resistance genes. *J Antimicrob Chemother* 67**,** 2640-2644.

Zhang, S., Den Bakker, H.C., Li, S., Chen, J., Dinsmore, B.A., Lane, C., Lauer, A.C., Fields, P.I., and Deng, X. (2019). SeqSero2: Rapid and Improved Salmonella Serotype Determination Using Whole-Genome Sequencing Data. *Appl Environ Microbiol* 85.
