# Supplementary material for: Monitoring the Microevolution of Salmonella enterica in Healthy Dairy Cattle Populations at the Individual Farm Level Using Whole-Genome Sequencing
Source: Front Microbiol. 2021 Oct 18;12:763669. doi: 10.3389/fmicb.2021.763669 (PMC8558520; doi:10.3389/fmicb.2021.763669)
Supplement: Supplementary file 7 [file Image_1.PDF]

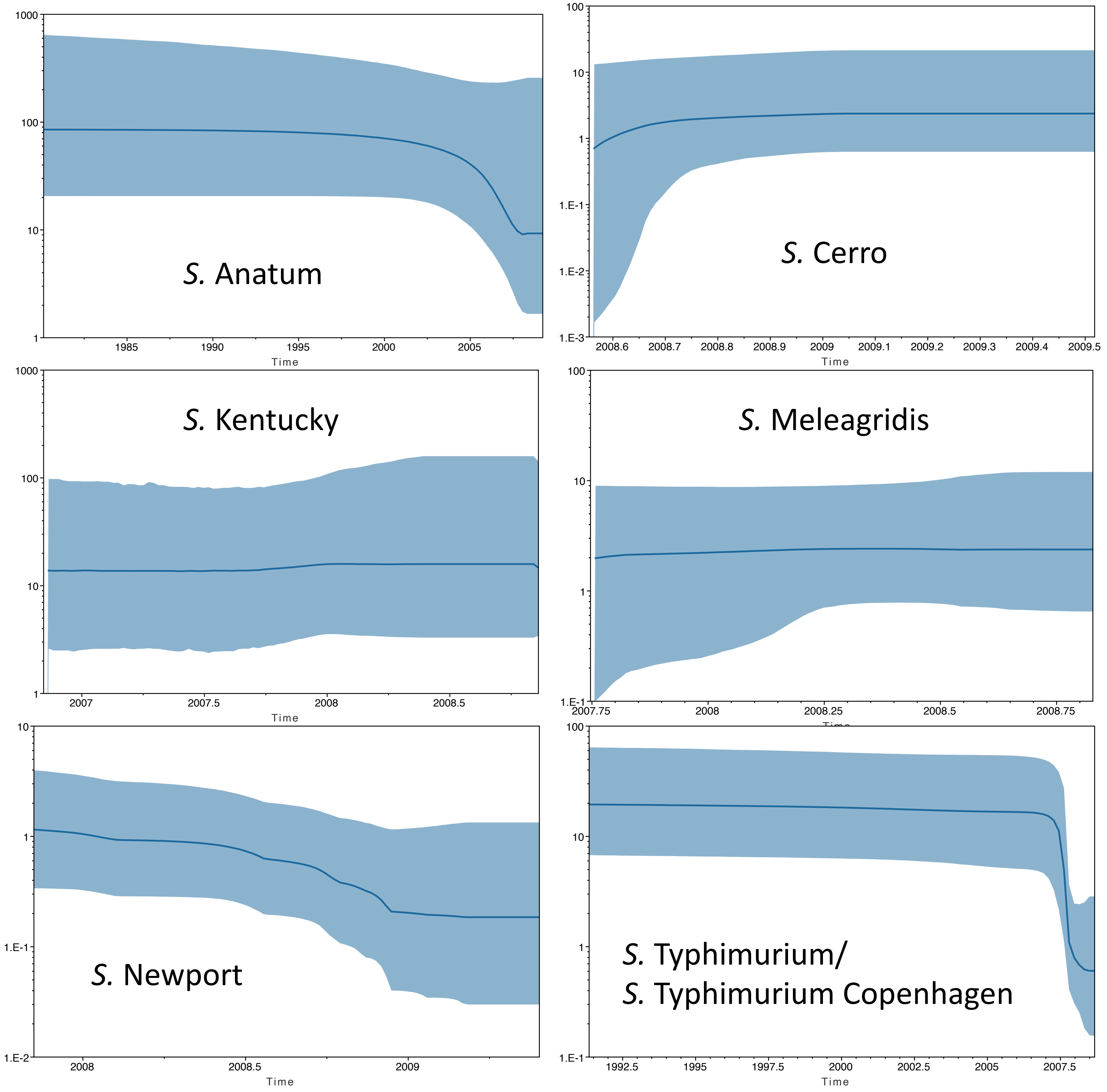

Supplementary Figure S1. Skyline plots constructed for each *Salmonella* serotype group. Effective population size and time in years are plotted on the Y- and X-axes, respectively. The median effective population size estimate is denoted by the blue line, with upper and lower 95% highest posterior density interval bounds denoted by blue shading.
